# Draft Genome Sequences of Four *Xanthomonas arboricola* pv. juglandis Strains Associated with Walnut Blight in Chile

**DOI:** 10.1128/genomeA.01160-15

**Published:** 2015-10-08

**Authors:** Gastón Higuera, Narjol González-Escalona, Camila Véliz, Francisca Vera, Jaime Romero

**Affiliations:** a Laboratorio de Biotecnología, Instituto de Nutrición y Tecnología de los Alimentos, Universidad de Chile, Macul, Santiago, Chile; b Division of Microbiology, Center for Food Safety and Applied Nutrition, Food and Drug Administration, College Park, Maryland, USA

## Abstract

*Xanthomonas arboricola* pv. juglandis is an important pathogen responsible for walnut blight outbreaks globally. Here, we report four draft genome sequences of *X. arboricola* pv. juglandis strains isolated from Chilean walnut trees.

## GENOME ANNOUNCEMENT

The Persian or English (*Juglans regia* L.) walnut tree is a species widely cultivated worldwide. Walnut blight is the main disease affecting walnut production; if not controlled, yield losses can exceed 50% (1, 2). It is caused by the bacterium *Xanthomonas arboricola* pv. juglandis (Xaj); Xaj is especially important in locations that have warm and rainy springs, because they are favorable conditions for rapid proliferation (2, 3). In Chile, walnuts tree are planted from the Copiapó (latitude 27°30′S) down to Temuco (38°45′S), with Xaj affecting the southern areas of the country.

Agrochemicals based on copper in its various formulations are the principal tools used in controlling Xaj in walnut trees (4). Despite its widespread use, its efficiency has decreased, which could be explained by the ability of bacteria to acquire resistance mechanisms (4–6). This situation is aggravated by evidence suggesting the transfer of copper resistance genes between bacteria (5). The analysis of the four genomes of Xaj presented here could help researchers understand the mechanisms involved in copper resistance in this pathogen.

Genomic DNA of each Xaj strain was extracted using the QIAamp DNA minikit (Qiagen, Valencia, CA) according to the manufacturer’s protocol. Libraries were prepared using 1 ng of genomic DNA with the Nextera XT kit (Illumina, San Diego, CA), and the genomes were sequenced using MiSeq Illumina with the V2 kit (2 × 250 bp) according to the manufacturer’s instructions at 90× to 160× coverage. Genomic sequence contigs for each strain were *de novo* assembled using CLC Genomics Workbench version 8.2 (Qiagen). The genomic analysis was performed using the RAST server (7). The assembled sequences were annotated by the National Center for Biotechnology Information (NCBI) Prokariyotic Genomes Annotation Pipeline (PGAP, http://www.ncbi.nlm.nih.gov/genome/annotation_prok). The summary report for the 4 genomes sequenced in this study (genome size, G+C content, contigs number, and the number of RNA coding genes) are in Genes related to copper resistance mechanisms (*copA, copB, copD, copG, cusA, cusC, cusF, cutA,* and *cutC*) were detected in the four draft genomes, their similarity in nucleotide-based comparison ranged 86 to 100%. A detailed report of genomic features will be addressed in a future publication.

### Nucleotide sequence accession numbers.

Genomes were deposited DDBJ/EMBL/GenBank under the accession numbers listed in Table 1. The assembly versions described in this paper are the first version of the assemblies.

**TABLE 1:** Summary report of the *de novo* assembly of the four Chilean *Xanthomonas arboricola* pv. juglandis strains from this study

Strain	CFSAN no.	GenBank accession no.	% G+C content	Genome size (bp)	No. of contigs	Avg coverage	No. of tRNAs	No. of rRNAs
Xaj2	CFSAN033077	LHBK00000000	65.4	5,101,226	197	150×	51	3
Xaj43a	CFSAN033086	LHBT00000000	65.4	5,144,142	205	90×	51	3
XajA3	CFSAN033085	LHBS00000000	65.6	5,118,988	196	168×	51	3
Xaj4.1	CFSAN033078	LHBL00000000	65.6	5,116,263	215	107×	49	3
